# Calcium oxalate crystal related kidney injury in a patient receiving Roux-en Y hepaticojejunostomy due to gall bladder cancer

**DOI:** 10.1186/s12882-017-0520-y

**Published:** 2017-03-29

**Authors:** Jun-Li Tsai, Shang-Feng Tsai

**Affiliations:** 1 Department of Family Medicine, Cheng Ching General Hospital, Taichung, Taiwan; 2 Division of Nephrology, Department of Internal Medicine, Taichung Veterans General Hospital, 1650 Taiwan Boulevard Sect. 4, Taichung, Taiwan 40705 Taiwan, Republic of China; 3 Department of Life Science, Tunghai University, Taichung, Taiwan; 4 School of Medicine, National Yang-Ming University, Taipei, Taiwan

**Keywords:** Calcium oxalate, Acute kidney injury, Roux-en Y hepaticojejunostomy

## Abstract

**Background:**

Calcium oxalate nephropathy is rare in current practice. It was a common complication during jejunoileal bypass, but much less seen in modern gastric bypass surgery for morbid obesity. The major cause of it is enteric hyperoxaluria.

**Case presentation:**

We report on a patient here with acute kidney disease due to calcium oxalate nephropathy, rather than the conditions mentioned above. The male patient received a Roux-en Y hepaticojejunostomy and common bile duct drainage. In addition to enteric hyperoxaluria, chronic kidney disease related metabolic acidosis, chronic diarrhea related volume depletion, a high oxalate and low potassium diet, long term ascorbic acid intake and long term exposure to antibiotics, all predisposed him to having oxalate nephropathy.

**Conclusion:**

This is the first case with such conditions and we recommend that similarly diagnosed patients avoid all these predisposing factors, in order to avoid this rare disease and its undesired outcome.

## Background

Oxalate nephropathy is a rare disease, and most cases are due to enteric hyperoxalauria. Calcium oxalate crystal related renal injury in the past was mostly due to a jejunoileal bypass (JIB) performed for the treatment of obesity. Owing to too many complications, modern surgery for morbid obesity today is through Roux-en-Y gastric bypass (RYGB) surgery. Much less oxalate nephropathy will be seen in the currently performed surgery for morbid obesity. Herein, we report the first case of oxalate nephropathy related acute kidney injury receiving a Roux-en Y hepaticojejunostomy due to gall bladder carcinoma, not previously reported in the literature. We also did literature reviews for calcium oxalate nephropathy and held discussions regarding the characteristics of this patient.

## Case presentation

**Fig. 1:**
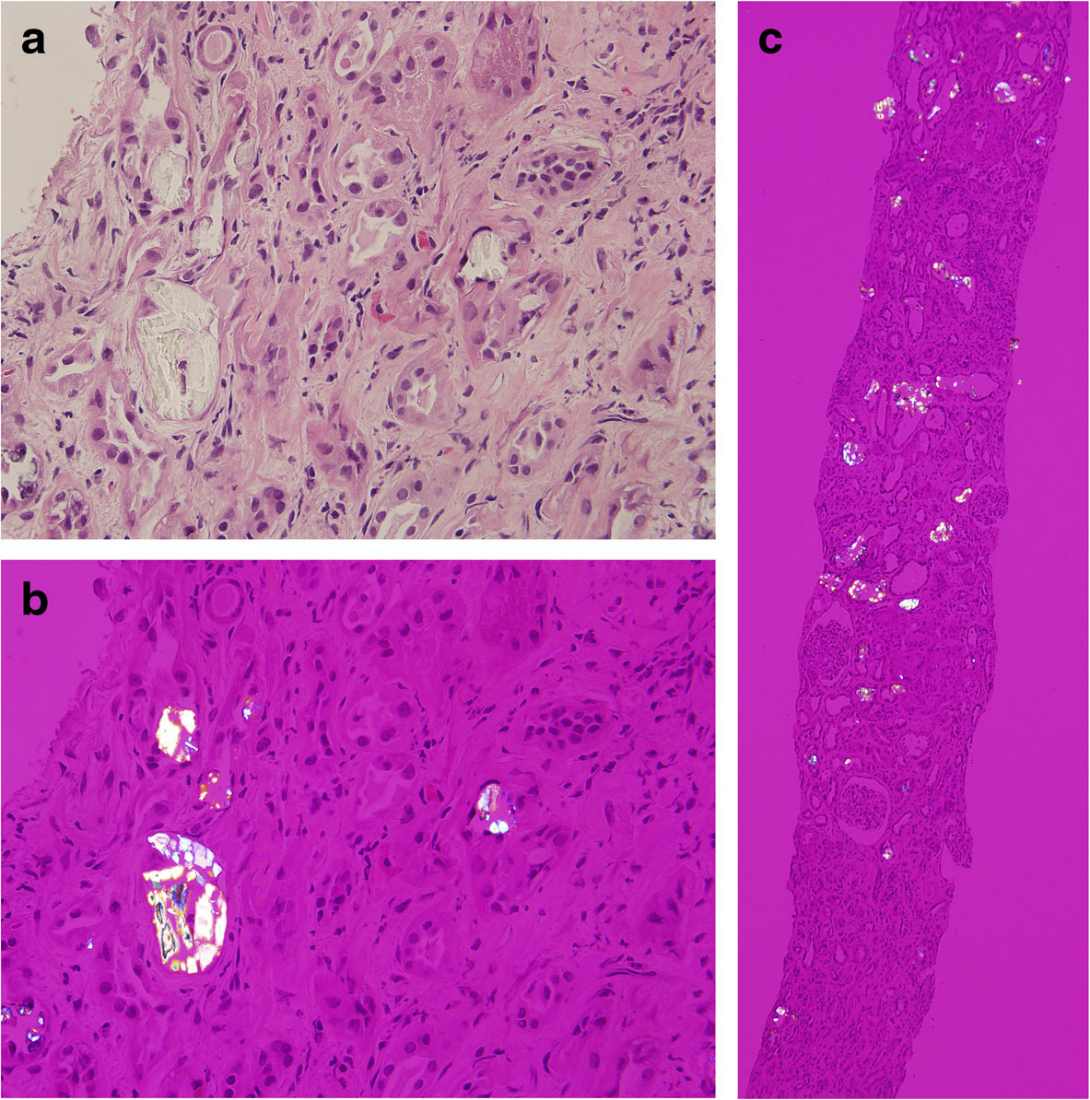
**a** Mild interstitial inflammation, moderate interstitial fibrosis and tubular atrophy with intratubular crystal deposition. (400X, PAS staining). **b** The deposits appeared strongly birefringent, forming fan-like, sheaf-like, or irregular shapes consistent with calcium oxalate crystals (400X, polarized light). **c** Almost all tubular lumens were filled with crystals (100X, polarized light)

A 67-year-old man was experiencing gallbladder carcinoma with common bile duct (CBD) invasion related jaundice and cholangitis s/p cholecystectomy, hilar lymph node dissection and Roux-en Y hepaticojejunostomy three years ago. However, six months after diagnosis, the carcinoma reoccurred, so he received a partial hepatectomy along with chemotherapy (Cisplatin and 5-FU over six cycles). After chemotherapy, the serum creatinine was 4.7 mg/dl (13 mL/min/1.73 m^2^ of GFR). Due to obstructive jaundice, a CBD drainage tube was inserted. He also experienced frequent cholangitis and his bile cultures yielded *Klebsiella pneumoniae, Citrobacter freundiim, Enterococcus sp., Group D streptococcus not enterococcus, and Escherichia coli*. Due to these conditions, he received Cefoperazone for an extensive period of time (6 months). Afterward, he suffered from chronic diarrhea for two years, five times per day. The renal function was stable even the above conditions. This time (three years after RYGB), he was admitted to hospital due to kidney injury (4.7 to 14.7 mg/dl of serum creatinine), which is less likely pre-renal azotemia. A renal biopsy disclosed mild interstitial inflammation, moderate interstitial fibrosis and tubular atrophy with intratubular crystal deposition (Fig. 1a). Under polarized light, the deposits appeared strongly birefringent, which formed fan-like, sheaf-like, or irregular shapes consistent with calcium oxalate crystals (Fig. 1b). Under a low power field with polarized light, almost all tubular lumens were filled with crystals (Fig. 1c). The patient’s 24-h urinary oxalate level was found to be significantly elevated, at 60 mg/day (normal range is up to 40 mg/day). Serum calcium, thyroid function, and uric acid were all within normal limits. Calcium oxalate crystal related acute kidney injury was confirmed, and the patient was placed on a low oxalate diet. However, soon after the diagnosis, he went into end-stage renal disease and was placed on hemodialysis. Before this admission, he had been on a low potassium diet because of long-term renal dysfunction. The patient himself had approved the course of treatment, and had given signed consent.

## Discussion

Endogenous oxalate is primarily derived from the metabolism of glycine and ascorbic acid [1]. Calcium oxalate crystal related renal injury in the past was mostly due to JIB, which was a surgical weight-loss procedure performed for the relief of morbid obesity during the 1950s through the 1970s. However, there remained too many complications, including mineral and electrolyte imbalance, protein calorie malnutrition, enteric complications, and renal disease (hyperoxaluria, with oxalate stones or interstitial oxalate deposits). The multiple complications which were associated with JIB, led to a search for alternative procedures. In 1979 [2], one type of modern bariatric surgery was gastric bypass. Full procedure gastric bypass included RYGB surgery, biliopancreatic diversion with duodenal switch, vertical banded gastroplasty, and laparoscopic adjustable gastric banding. A study by Asplin et al. revealed a mean oxalate excretion of 83 mg/day in patients who underwent RYGB [3]. This was significantly higher than that found in routine kidney stone formers or normal subjects (39 and 34 mg/day, respectively), but lower when compared to patients who had undergone JJIB (103 mg/day). To the best of our knowledge, our patient is the first case of a calcium oxalate related renal injury due to having received a Roux-en-Y hepaticojejunostomy, in order to treat gallbladder carcinoma rather cancer obesity.

The cause of hyperoxaluria related calcium oxalate crystals could be multifactorial in nature. Firstly, malabsorption leading to steatorrhea plays a central role in all forms of enteric hyperoxaluria [4, 5]. Under normal circumstances, calcium will bind to oxalate within the intestinal lumen to form insoluble calcium oxalate, that is excreted in feces. If with fat malabsorption, excessive intraluminal free fatty acids bind to and saponify calcium, which inhibits the formation of calcium oxalate. Bile plays an important role in fat digestion, emulsifying fat as the first stage in its digestion. There was only a 50% bile level for this patient to help digest fat because a CBD drainage was performed to avoid obstructive jaundice (about 500 ml per day, according to the medical records). Therefore, because fat malabsorption was anticipated in this patient, more soluble free oxalate was absorbed by the colonic mucosa. In addition, the other 50% nonabsorbed bile salts were drained into the colon, causing increased colonic permeability to small molecules such as oxalate [6, 7]. Both soluble free oxalate in the lumen, and higher permeability to oxalate cause enteric hyperoxaluria. Secondly, the patient also experienced diarrhea without enough fluid supplements to enable him to avoid chronic kidney injury related edema. The diarrheal fluid losses can lead to a reduction in urine volume, along with intratubular concentrations of oxalate and calcium.

In addition to the risk factors associated with the surgery or bile salt, the patient also had other predisposing factors. One is that he had renal dysfunction related metabolic acidosis, which led to a low urine pH (5.0). Since acidemia enhances proximal citrate reabsorption [8] and urinary citrate is a potent inhibitor of calcium stone formation, these factors promote calcium oxalate precipitation. Secondly, the patient changed his dietary intake habits due to long-term diarrhea. He consumed food with high oxalate levels each day, including beans, beets, cranberries and nuts. He also thoroughly enjoyed both coffee and black tea. Therefore, his diet contained high oxalate levels. Thirdly, prolonged administration of antibiotics will cause loss of *Oxalobacter formigenes*, which can lead to a decreased degradation of oxalate, and thus, increasing the risk of hyperoxaluria [9]. Fourthly, as a patient with chronic kidney disease, he maintained a low potassium diet. However, it is a higher potassium intake that may reduce the risk of stone formation by reducing urinary calcium excretion [10], and may also increase urinary citrate excretion, because potassium-rich foods tend to have a high alkali content. Thus, his low potassium diet further made the patient more susceptible to calcium oxalate nephropathy. Fifthly, the patient had been taking ascorbic acid for two years to assist in the healing of a scar, which was due to earlier surgery and a CBD drainage catheter. A high dosage of vitamin C could cause an increase in oxalate generation [11]. Last but not the least, Cisplatin may enhance calcium oxalate crystal adherence to inner medullary collecting duct cells [12] even though the contribution of Cisplatin to oxalate nephropathy is more speculative than other causes.

In a series of 132 patients with nephrolithiasis who had undergone gastric bypass surgery, the average time for the formation of the first stone was 3.6 years [3], which is compatible with our case. Even though his urinary oxaluria was much less than patients undergoing gastric bypass (60 mg/day vs. 83 mg/day), this patient still had other risk factors involved, including metabolic acidosis, chronic diarrhea related volume depletion, high oxalate and low potassium diet, long-term ascorbic acid intake, along with long-term exposure to antibiotics.

The prognosis of oxalate nephropathy after RYGB would be considered dismal, with the progression to end-stage renal disease coming within three months in 72.7% of the patients in this study [13]. In our case, the prognosis of oxalate nephropathy after Roux-en Y hepaticojejunostomy was also poor. The patient required long-term hemodialysis after this episode of acute kidney injury, even while maintaining a low oxalate diet.

This is a limitation to this report because we did not have the report of citrate, calcium, and uric acid of 24-h urine study.

## Conclusion

In patients receiving Roux-en Y hepaticojejunostomy, it is necessary to keep in mind the possibility of oxalate nephropathy occurring. Avoiding volume depletion, renal dysfunction, long-term antibiotics, Vitamin C and a low potassium intake are all mandatory guidelines in order to help prevent this rare disease and its undesired outcome.

## Abbreviations

CBD: Common bile duct
JIB: Jejunoileal bypass
RYGB: Roux-en-Y gastric bypass
